# Hirschsprung Disease: A Literacy Analysis of Patient Information

**DOI:** 10.7759/cureus.46806

**Published:** 2023-10-10

**Authors:** April L Baum, Anh Nguyen, Michael J Valentine, Larissa Vollin, Caleb R Mcnab, Carol E Kirila

**Affiliations:** 1 Pediatrics, Kansas City University, Kansas City, USA; 2 Primary Care/Internal Medicine, Kansas City University, Kansas City, USA; 3 Internal Medicine, Kansas City University, Kansas City, USA

**Keywords:** click-through rate, aganglionic megacolon, hirschsprung disease, online information, readability, health literacy

## Abstract

Objective: Hirschsprung disease in newborns can be a potentially life-threatening condition, with risks for complications such as Hirschsprung-associated enterocolitis. Accessing health information in a readable format for complex diseases demonstrates an important outlet for families to address concerns. While it is important to seek out information from trusted providers, many individuals seek out ways to educate themselves further by using common search engines and turning to the internet. This article will evaluate the readability of relevant articles on Hirschsprung disease and information accessibility to the average health literacy individual.

Methods: A readability analysis of the first 20 Google search results from the keywords “Hirschsprung disease” and “aganglionic megacolon” was performed. Results were documented and averaged using standardized scoring systems. Scoring systems included an Automated Readability Index, Coleman Liau index, SMOG index, Gunning Fog score, Flesch Kinkaid grade level, Flesch Kinkaid reading ease, and average readability across all scoring systems. The number of sentences, number of words, number of complex words, percent of complex words, average words per sentence, and average syllables per word were included as a sample of criteria within scoring systems.

Results: The average readability score for the first 20 search results of keywords “Hirschsprung disease” and “aganglionic megacolon” was 9.4, indicating a reading level just above the ninth grade. Readability ease was scored separately due to its unique scoring system on a scale of 0-100. Flesch Kincaid Readability ease score was averaged and resulted in a score of 46.4, which is considered “college level” or “difficult.”

Conclusions: The average health consumer will turn to Google to find information about their own health, as well as the health of their loved ones. Hirschsprung disease in newborns presents a complex disease process and can be potentially life-threatening. Current resources available to the average health consumer averaged at a grade level of 9.4 relative to search results from keywords “Hirschsprung disease” and “aganglionic megacolon.” Depending on the target audience for online information, more work needs to be done to improve readability for the average health information consumer.

## Introduction

Hirschsprung disease affects about one in 5,000 newborns [1]. This complex disease process involves the improper migration of neural crest cells during development, resulting in absent myenteric (Auerbach) and submucosal (Meissner) plexus ganglion nerve cells in the distal aspect of the digestive tract. A heightened concern for Hirschsprung disease should be considered when an infant fails to pass meconium within the first 24-48 hours after birth [2].

Diagnosis of this condition often involves a detailed medical and family history, imaging, anorectal manometry, and/or biopsy. The most conclusive test to confirm Hirschsprung disease is to perform rectal suction vs. full-thickness rectal biopsy [3]. A pathologist review of the tissue with positive confirmation will demonstrate a lack of ganglion nerve cells. Infants with this condition are at risk of Hirschsprung-associated enterocolitis, a potentially life-threatening and severe complication.

For any new parents experiencing a life-threatening condition in their newborn, the readability of information is relevant and crucial to address concerns and ensure an accurate understanding of the material. According to the National Literacy Institute, 54% of adults in the US have a literacy below a fifth-grade reading level [4,5]. For complex disease processes, patient education materials need to use clear, concise language at a reading level that is accessible to the average health literacy patient.

## Materials and methods

Evaluating readability

Readability analyses are useful to measure the level needed to comprehend a unit of written text. They have been utilized many times in the medical field [6-11]. Google search for “Hirschsprung disease” and “aganglionic megacolon” was conducted, and the first 20 websites in the English language were collected as data. A total of 38 websites were included in the final data analysis. Sponsored content and videos were excluded, as well as sources that require payment to access. Additionally, there was no vetting for the credibility of source content upon choosing data for readability analysis. The websites used are displayed in Table 1.

**Table 1:** Websites used for key search criteria. UpToDate websites (bolded in both columns) were excluded due to paid content and restricted public access.

Position in Query	Google search 1: "hirschsprung's disease"	Google search 2: "aganglionic megacolon"
1	https://www.niddk.nih.gov/health-information/digestive-diseases/hirschsprung-disease	https://www.childrenshospital.org/conditions/hirschsprungs-disease
2	https://www.mayoclinic.org/diseases-conditions/hirschsprungs-disease/symptoms-causes/syc-20351556	https://www.mayoclinic.org/diseases-conditions/hirschsprungs-disease/symptoms-causes/syc-20351556
3	https://www.childrenshospital.org/conditions/hirschsprungs-disease	https://www.uptodate.com/contents/congenital-aganglionic-megacolon-hirschsprung-disease
4	https://medlineplus.gov/genetics/condition/hirschsprung-disease/	https://medlineplus.gov/genetics/condition/hirschsprung-disease/
5	https://www.chop.edu/conditions-diseases/hirschsprung-s-disease	https://www.ncbi.nlm.nih.gov/medgen/5559
6	https://rarediseases.org/rare-diseases/hirschsprungs-disease/	https://www.cincinnatichildrens.org/health/h/hirschsprung
7	https://www.nationwidechildrens.org/conditions/hirschsprung-disease	https://www.chop.edu/conditions-diseases/hirschsprung-s-disease
8	https://emedicine.medscape.com/article/178493-overview	https://www.ncbi.nlm.nih.gov/books/NBK562142/
9	https://www.cincinnatichildrens.org/health/h/hirschsprung	https://emedicine.medscape.com/article/178493-overview
10	https://my.clevelandclinic.org/health/diseases/9844-hirschsprung-disease	https://www.health.state.mn.us/diseases/cy/hirschsprung.html
11	https://kidshealth.org/en/parents/hirschsprung.html	https://accesspediatrics.mhmedical.com/content.aspx?bookid=2196&sectionid=166956568
12	https://www.hopkinsmedicine.org/health/conditions-and-diseases/hirschsprungs-disease	https://jamanetwork.com/journals/jamapediatrics/fullarticle/498264
13	https://www.nhs.uk/conditions/hirschsprungs-disease/	https://www.darmzentrum-bern.ch/fileadmin/darmzentrum/Education/Bible_Class/2021/Colonic_Motility_Disorders/2._Congenital_aganglionic_megacolon__Hirschprung_disease_.pdf
14	https://en.wikipedia.org/wiki/Hirschsprung%27s_disease	https://www.sciencedirect.com/topics/medicine-and-dentistry/hirschsprungs-disease
15	https://www.webmd.com/children/what-is-hirschsprungs-disease	https://www.britannica.com/science/congenital-megacolon
16	https://www.stanfordchildrens.org/en/topic/default?id=hirschsprungs-disease-90-P01999	https://link.springer.com/chapter/10.1007/978-3-319-06665-3_32
17	https://www.chp.edu/our-services/surgery-pediatric/pediatric-surgery-services-we-offer/colorectal-center-for-children/conditions-we-treat/hirschsprungs-disease	https://www.cancertherapyadvisor.com/home/decision-support-in-medicine/critical-care-medicine/gastrointestinal-emergencies-hirschsprung-disease-congenital-aganglionic-megacolon/
18	https://www.stlouischildrens.org/conditions-treatments/hirschsprung-disease	https://www.chp.edu/our-services/transplant/intestine/education/intestine-disease-states/hirschprungs-disease
19	https://www.uptodate.com/contents/congenital-aganglionic-megacolon-hirschsprung-disease	https://teachmepaediatrics.com/surgery/abdominal/hirschsprungs-disease/
20	https://surgery.ucsf.edu/conditions--procedures/hirschsprungs-disease.aspx	https://medical-dictionary.thefreedictionary.com/aganglionic+megacolon

Google search keywords

To select the Google search keywords, the pathophysiology of the disease state itself was considered. Two keywords were developed in order to account for the variabilities in the patient search for Hirschsprung disease. These differences in patterns of search could be based on educational background and exposure to different references of the disease by physicians and healthcare workers. Google was the most utilized search engine due to its popularity in the United States, being the engine that was used 85% of the time by the average American [12].

Data and readability analysis

WebFX is a readability analysis tool that is highly recommended by the Search Engine Journal [13]. This tool generates readability scores across different indices, which place particular emphasis on different areas of a URL website in order to determine a score. The following indices were used in data analysis: Automated Readability Index (ARI), Coleman-Liau Index (CLI), Simple Measure of Gobbledygook (SMOG), Gunning Fox Index (GFI), and Flesh-Kincaid Grade Level (FKGL). Readability indices with measurement considerations are displayed in Table 2. The scores from each index were computed into averages and standard deviations based on its respective groups of Google searches, from the “Hirschsprung disease” and “aganglionic megacolon” groups, and subsequently analyzed to determine readability by the general public.

**Table 2:** Readability indices with corresponding measurement considerations. Each index uses grade level as a unit of results. Note that the Flesch Kincaid Reading Ease utilizes a different scoring system, in which case the scale is from 0 to 100 (*). Abbreviations: SMOG - simple measure of gobbledygook, US - United States

Readability Indices [13-14]	Measurement Considerations	Reading Level Interpretation
Flesch Kincaid Grade Level	Sentence length and number of syllables per sentence	US Grade levels (5th-grade to college graduate)
Gunning Fog Index	Numbers, length, and complexity of sentence	US Grade levels (5th-grade to college graduate)
SMOG Index	Amount of complex word density of sentence	US Grade levels (5th-grade to college graduate)
Coleman Liau Index	Number of characters and words per sentence	US Grade levels (5th-grade to college graduate)
Automated Readability Index	Characters and sentence length	US Grade levels (5th-grade to college graduate)
Flesch Kincaid Reading Ease	Words per sentence and syllables per word	0 - 100*

The Flesch Kincaid Reading Ease utilizes a score output of zero to 100. The higher the score, the better the readability and thus the easier it is to read. While this reading index may have a simple scoring output, it may be difficult to comprehend what each score means. A score range of zero to 30 indicates a college graduate reading level, whereas a score range of 90 to 100 indicates a US 5th-grade reading level [8]. Table 3 contains an interpretation of the Flesch Kincaid Reading Ease scores.

**Table 3:** Scoring scale used to interpret a Flesch Kincaid Reading Ease score. Notably, this reading index uses an output that is not directly related to US grade reading levels. This is different from most other reading indices. Abbreviations: US - United States

Flesch Kincaid Reading Ease Score [14]	US Grade Reading Level
0 - 30	College Graduate
30 - 50	College Undergraduate
50 - 60	10th - 12th-Grade
60 - 70	8th - 9th-Grade
70 - 80	7th-Grade
80 - 90	6th-Grade
90 - 100	5th-Grade

Health-specific click-through rate (CTR)

The CTR is the rate at which a website is clicked on when a user searches for a query on the search engine. This value is considered a predictive value, which varies based on which keywords were used to look up a topic, which device they used, and where the user was located [6,15]. Regardless of the variability, it is commonly accepted that the higher a CTR value is, the more impressions the URL has made and, therefore, attains a higher quality score, equating to higher viewership. The scores can be relative, for each industry has typical average scores that vary. The most relevant category for this study is considered “Health and Fitness”, which has an average CTR of 6.15%, whereas other categories, such as “Travel” and “Arts and Entertainment,” have an average CTR of 9.19% and 11.43%, respectively [15].

## Results

**Figure 1:**
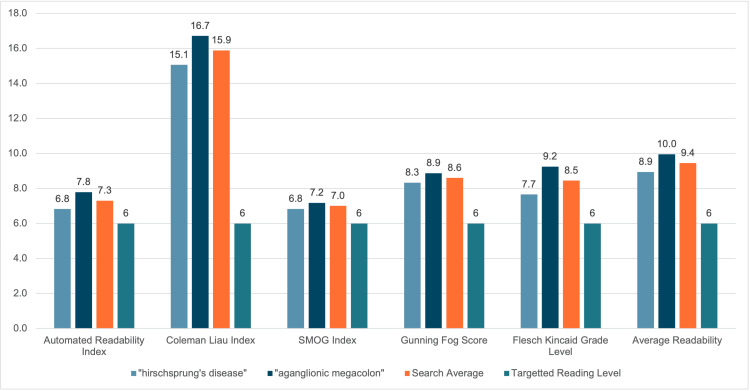
Readability indices and averages of key search criteria, juxtaposed with calculated combined average of both keyword search index results. The sixth-grade reading level is also shown for comparison, representing the NIH and AMA recommended reading level for patient education materials. Abbreviation: SMOG - Simple Measure of Gobbledygook

The total average readability score for the search keywords “Hirschsprung disease” and “aganglionic megacolon” was 9.4, equating to a reading level just above the ninth grade. The combined average readability score of “Hirschsprung disease” yielded 8.9, whereas the combined average readability score of “aganglionic megacolon” yielded 10. A breakdown of the average of each unique keyword search relative to the Automated Readability Index, Coleman Liau Index, SMOG Index, Gunning Fog Score, and the Flesch Kincaid Grade Level, may be found in Figure 1.

These readability indices have been utilized in various medical fields as an objective means of text comprehension measurement [6-11]. Notably, two websites required payment for access. These websites were UpToDate links, which are commonly used as clinical aid resources for physicians and mid-levels. Many patients will not have the background or means to comprehend a resource that is used by clinicians past the college graduate level. Thus, links to websites that are used as a source for medical professionals are inaccessible to the general public.

The Flesch Kincaid Reading Ease average was calculated separately, because it uses a scale of 0-100, rather than basing results on US grade levels. The Flesch Kincaid Reading Ease yielded an average of 46.4, which falls into the category of “difficult” or “college level” (Table 3). The college-level score of reading ease is considered within a score range of 30-50 (Table 3). These results included 38 of the intended 40 websites that were analyzed due to exclusion criteria (Table 1). Exclusion criteria included required payment or lack of accessibility to the public.

## Discussion

It is widely observed that individuals frequently turn to search engines like Google to access health-related information. While this practice can certainly enable people to broaden their understanding of medical issues, it also carries inherent risks. Specifically, if the information obtained is either misunderstood or misinterpreted, it may lead to complications for those who are seeking medical advice. This becomes particularly alarming when patients and their families rely on such online resources as a basis for making critical medical decisions.

According to data from the National Literacy Institute, a staggering 54% of adults in the United States possess literacy skills that are below a sixth-grade level [5]. This literacy deficit not only has individual implications but also carries significant economic ramifications. Specifically, it is estimated that the societal cost of illiteracy to American taxpayers amounts to approximately $20 billion annually. Transitioning from the economic sphere to the criminal justice system, another disconcerting observation emerges. Some states employ predictive models for future prison bed requirements that incorporate reading assessment scores from elementary school students [5]. This practice underscores the long-term societal consequences of inadequate literacy skills, extending beyond immediate educational concerns. In the healthcare sector, the issue of literacy takes on a daily challenge. Estimates suggest that nearly 50% of Americans have reading skills so limited that they may struggle to comprehend the instructions on their prescription bottles [5]. In a generation filled with copious online information, it becomes increasingly evident that literacy - or the lack thereof - substantially influences the quality of life of the general population.

Our results showed an average readability score above the ninth grade related to search results on the topic of Hirschsprung disease. The Flesch Kincaid Reading Ease demonstrated a score correlating with college-level reading material falling into the category of “difficult.” The five other readability indices demonstrated a total average of 9.4, suggesting, at the minimum, requiring a 9th-grade reading level to understand online information regarding Hirschsprung disease.

This finding is particularly concerning when considered in the context of teenage mothers. A study performed in 2018 found that approximately 47% of US teenage mothers did not complete high school [16]. Furthermore, In 2012, US. teen births constituted 7.8% of all births and 17.1% of all non-marital births [17]. Given the established correlation between teenage motherhood and adverse health outcomes for both mother and child [17], the inaccessibility of online medical information exacerbates these challenges. Existing literature suggests that patient outcomes improve significantly when complex medical topics are distilled to a level that is understandable to the patient [18].

According to the Minnesota Department of Health, 20% of neonatal bowel obstructions can be attributed to Hirschsprung disease [19]. For parents of newborns experiencing this complex and potentially severe disease, online health information correlates with a challenging reading level. This emphasizes the importance of effective provider communication to ensure that families have a clear understanding of relevant health information. Additionally, the NIH and AMA recommend that patient education materials are at or below a 6th-grade reading level [6,20]. While target audiences for online information may vary, it is important to increase access to information that is comprehensible to the average individual. This will ensure that a broader demographic may benefit from critical health information, thereby potentially improving patient outcomes.

Limitations

Data was excluded from the analysis if payment was required or if the resource was not available to the general public, resulting in 38 total website analyses, rather than the intended 40. Furthermore, given that this analysis is employed by a scoring system, the results may vary if the material is given access to parents, and then their understanding is further checked by the “repeat back” system. Sponsored content and video sources were also excluded. Source content was not vetted for credibility, which could be evaluated in future studies. This would be relevant to the average health information consumer, not only for the ability to understand content but also for the ability to exclude data without evidence-based information. In hindsight, a survey targeted at a pediatric clinic and parents specifically, would have been beneficial in rationalizing the two unique keyword searches. Additionally, this analysis is subject to change as time moves forward and internet traffic is altered from Google.

## Conclusions

These results show that the average individual searching for information on Hirschsprung disease with keywords “Hirschsprung disease” and “aganglionic megacolon” averaged 3.4-grade levels above the recommended health information level of sixth grade (average readability of both keyword searches is 9.4). Online sources providing public information on Hirschsprung disease targeting the public need to consider appropriate readability. While this is important for online health information due to the popularity of sources such as Google, this is especially important for official patient education materials.
